# Postacute Sequelae of SARS-CoV-2 in University Setting

**DOI:** 10.3201/eid2903.221522

**Published:** 2023-03

**Authors:** Megan Landry, Sydney Bornstein, Nitasha Nagaraj, Gary A. Sardon, Amanda Castel, Amita Vyas, Karen McDonnell, Mira Agneshwar, Alyson Wilkinson, Lynn Goldman

**Affiliations:** The George Washington University Milliken Institute School of Public Health, Washington DC, USA

**Keywords:** COVID-19, postacute sequelae, coronavirus disease, long COVID, severe acute respiratory syndrome coronavirus 2, SARS-CoV-2, college, university, students, young adult, members, infectious disease, respiratory infection, prevention, school health, outbreak prevention, contact tracing, pandemic, zoonoses, United States

## Abstract

Postacute sequelae of SARS-CoV-2 infection, commonly known as long COVID, is estimated to affect 10% to 80% of COVID-19 survivors. We examined the prevalence and predictors of long COVID from a sample of 1,338 COVID-19 cases among university members in Washington, DC, USA, during July 2021‒March 2022. Cases were followed up after 30 days of the initial positive result with confidential electronic surveys including questions about long COVID. The prevalence of long COVID was 36%. Long COVID was more prevalent among those who had underlying conditions, who were not fully vaccinated, who were female, who were former/current smokers, who experienced acute COVID-19 symptoms, who reported higher symptom counts, who sought medical care, or who received antibody treatment. Understanding long COVID among university members is imperative to support persons who have ongoing symptoms and to strengthen existing services or make referrals to other services, such as mental health, exercise programs, or long-term health studies.

It is estimated that 1 in 3 Americans who have SARS-CoV-2 infection will experience symptoms related to postacute sequelae of SARS-CoV-2 (*1*), also referred to as long COVID (other terms include long-haul coronavirus disease, post–-COVID-19 conditions, or chronic COVID-19) (*2*). The length of time that a person must experience symptoms to be considered to have long COVID is not universally accepted; definitions range from 28 days to 6 months after acute SARS-CoV-2 infection (*3**–**7*). A recent World Health Organization working group used a Delphi process to conclude that “a post-COVID-19 condition occurs in individuals with a history of probable or confirmed SARS-CoV-2 infection, usually 3 months from the onset of COVID-19 with symptoms that last for at least 2 months and cannot be explained by an alternative diagnosis” (*8*).

Regardless of a universally agreed upon length of time a person must experience symptoms to be characterized as long COVID, this sequela has been suggested to be the “next national health disaster” (*9*), and because of discrepancies in symptoms and long-term effects on quality of life, there seem to be more questions than answers. Although long COVID manifests differently in each person, nearly 50 signs and symptoms have been linked to the condition (*10*). The most common signs and symptoms are fatigue, shortness of breath, muscle pain, joint pain, headache, cough, chest pain, altered smell, altered taste, and diarrhea (*11*). Other reported signs and symptoms include cognitive impairment (known as brain fog), memory loss, palpitations, anxiety, sore throat, sleep disorders, runny nose, sneezing, hoarseness, ear pain, thoughts of self-harm and suicide, seizures, and bladder incontinence (*8*,*11*), as well as cardiac effects, such as myocardial inflammation (*12*).

Although some investigators have reported that long COVID occurs at rates that are independent of symptom severity (*11**–**13*), others have found long COVID is more common among patients hospitalized for COVID-19 or those who experienced moderate-to-severe symptoms (*6*,*11*,*14*–*20*). However, long COVID has been observed in patients who were asymptomatic (*2*) or only experienced mild symptoms, and it has been reported that symptoms can fluctuate or relapse (*7**–**8*,*21**–**23*). Furthermore, little is known about long COVID signs and symptoms and predictors on a college campus, where most of the population is young and healthy, but among whom potential complications of long COVID could be detrimental to academic learning and overall quality of life.

Long COVID signs and symptoms might vary by sex, age, and initial illness severity. For example, nervous system symptoms such as headaches and dizziness are more common among women, but men are more likely to have musculoskeletal system symptoms such as pain in the muscles or joints and numbness of the limbs (*24*). Younger patients have reported more headaches, abdominal symptoms, and anxiety/depression, and older patients were more likely to have breathing difficulties, cognitive symptoms, pain, and fatigue (*19*).

Aside from the medical illness long COVID poses, persistent signs and symptoms can negatively affect leisure and work, causing further strain on one’s quality of life. Persons who have long COVID frequently experience a substantial reduction or impairment in the ability to engage in preillness levels of occupational, educational, social, or personal activities that persist for >6 months (*14*). They might also experience difficulty sticking to daily routines, dealing with stress, getting household tasks done, and caring for/supporting others (*25*). Abnormal scores on mental and cognitive health questionnaires have also been observed among patients who have long COVID (*7*). Our study builds on the existing knowledge base by examining the prevalence and predictors of long COVID among a sample of university members, including students, faculty, and staff, who tested positive for COVID-19 over an 18-month period.

## Methods

### COVID-19 Case Identification

The George Washington University COVID-19 surveillance and testing program identified 4,800 COVID-19 cases during August 2020‒February 2022. COVID-19 positivity at George Washington University was determined on the basis of PCR tests that were performed in the George Washington University Clinical Laboratory Improvement Amendments, or Clinical Laboratory Improvement Amendment‒certified, Public Health Laboratory (n = 3,228); other cases were identified through results uploaded to the person’s medical portal from external positive tests, either PCRs from an external Clinical Laboratory Improvement Amendment‒certified laboratory or self-administered antigen tests (n = 1,572). Only antigen tests approved for emergency use under the Food and Drug Administration emergency use authorization were accepted (*26*).

### COVID-19 Case Investigation Data Collection

As COVID-19 cases were identified, the George Washington University Campus COVID-19 Support Team (CCST), which is responsible for campus-related COVID-19 case management (*27*), completed case investigations within 24–48 hours of the person receiving a positive test result. Among the 4,800 positive results during August 2020‒February 2022, a total of 133 initial case investigations were incomplete because of loss to follow-up, meaning they could not be reached by telephone or electronic survey; because the case was already cleared by a medical provider (because of not being able to reach the person during their isolation period); or because the person refused to complete the interview. Furthermore, 1,072 persons were missing case investigation data, such as missing data for symptoms or underlying conditions, Those exclusions resulted in 3,595 positive test results (with corresponding completed case investigation data) for which CCST had obtained complete case investigation data (Table 1).

**Table 1:** Initial case investigation and long COVID data collection for postacute sequelae of SARS-CoV-2 in university setting, Washington, DC, USA*

Characteristic	Value
Positive test results reported during 2020 Aug–2022 Feb	4,800
GWU PCR	3,228
External CLIA PCR or self-administered antigen	1,572
Initial case investigation incomplete	133
Initial case investigation data missing	1,072
Total positive test results with case investigation data	3,595
Long COVID surveys sent out, 2021 Jul–2022 Mar	4,800
Repeat infection duplicates	143
Persons surveyed	4,657
Responses received	1,493
Duplicate responses/multiple responses	11
Persons responding	1,482
Response rate, 1,482/4,657	31.8%
Responders with incomplete case investigation	141
Responders with incomplete long COVID questions	3
Total valid responses	1,338

*Values are no. except as indicated. CLIA, Clinical Laboratory Improvement Amendments; GWU, George Washington University.

### Long COVID Follow-up Data Collection

During July 2021‒March 2022, all 4,800 positive COVID-19 test results reported during August 2020‒February 2022 were followed up with confidential electronic surveys sent to each patient at least 28 days after their initial positive result that included questions about long COVID. Those data were merged with the COVID-19 case investigation data.

For the long COVID follow-up survey data collection, we determined that 143 persons had >2 COVID-19 diagnoses during August 2020‒February 2022; those persons were only included once in the long COVID follow-up data collection, resulting in a total of 4,657 persons who were COVID-19 positive during the study period. The follow-up survey had a response rate of 32% (1,482/4,657). We observed major differences in age, university affiliation, underlying conditions, and vaccination status at the time of test between follow-up survey respondents and nonrespondents (Appendix 1). A total of 11 respondents completed the follow-up survey twice but were only counted once for the response rate. Not all responses were usable in the final analysis: 141 did not have a complete initial case investigation, and 3 did not provide responses to the survey questions about long COVID, removing them from the final sample. Thus, the final analytic sample consisted of 1,338 respondents (Table 1).

### Instrument and Measures

#### Survey Instrument

The long COVID survey was designed as a follow-up telephone interview (Appendix 2); interviews were administered by CCST during July 2021‒March 2022, and all survey responses were stored on REDCap, a secure web application for online surveys and databases (*28*). Initially, CCST interviewers exclusively administered the follow-up survey by telephone calls. However, after 3 months, a link to an electronic survey was sent to all remaining cases in addition to calling. Three call attempts were made over a period of 5 weeks, prompting case-patients to complete an anonymous survey. The long COVID survey consisted of close-ended questions pertaining to symptoms during the postisolation period and behavior changes from preisolation to postisolation periods (Appendix 1). The survey took ≈15–20 minutes to complete, and at the conclusion, a list of resources to assist with long COVID symptoms was provided.

#### Measures

We defined long COVID as experiencing >1 of the following symptoms lasting for >28 days after a respondent’s 10-day isolation period ended (*2*): difficulty driving, difficulty having conversations, difficulty making decisions, difficulty thinking, fatigue, feeling anxious, feeling depressed or sad, loss of smell, loss of taste, memory loss, muscle pain, muscle weakness, shortness of breath or difficulty breathing, trouble sleeping, worsening of symptoms after physical activity, worsening of symptoms after mental activity, or other symptoms. In addition, respondents were considered to have long COVID if they reported still experiencing COVID-19‒related symptoms at the time of the long COVID survey.

#### Sociodemographic Characteristics

We calculated age from the respondent’s date of birth extracted from their health record. Sex and race were self-reported at the time of the case investigation. We determined school affiliation by asking respondents their primary university affiliation at the time of the case investigation.

#### Symptoms and Underlying Conditions

We measured symptoms at the time of the case investigation by asking if respondents experienced any of the following: chest pain, chills, congestion, cough, diarrhea, fatigue, fever, headache, loss of smell, loss of taste, muscle pain, nausea or vomiting, runny nose, shortness of breath, sore throat, or other symptoms. At the time of the case investigation, respondents self-reported any of the following medical conditions: diabetes, asthma, hypertension, obesity, sickle cell disease, cancer, chronic kidney disease, lung diseases, serious heart conditions, or other conditions. Smoking status was self-reported as current/former smoker or vaper.

### Vaccination Status and Severity of COVID-19 Infection

Over the course of the study period, COVID-19 vaccine availability and recommendations shifted dramatically. In December 2020, vaccines were first available but only for select groups such as healthcare workers, the elderly, and certain other susceptible populations. During March‒April 2021, vaccines were made available to all adults (>16 years of age) across all US states. In June 2021, George Washington University mandated all members of the campus community to be up to date (an up-to-date course of COVID-19 vaccines consisted of either 2 doses of Moderna [https://www.modernatx.com] or Pfizer-BioNTech [https://www.pfizer.com] vaccines or 1 Johnson & Johnson/Janssen [https://www.jnj.com] immunization with the primary series of COVID-19 vaccinations or to have obtained an exemption. In September 2021, in the United States, COVID-19 booster shots were authorized for administration 6 months after the second dose of Pfizer or Moderna or 2 months after 1 dose of Johnson & Johnson/Janssen, initially just for persons >65 years of age, persons living or working in high-risk settings, or persons who had underlying conditions. In November 2021, booster shots were recommended for all adults >18 years of age. In January 2022, George Washington University mandated all members of the campus community to have a booster shot or to have obtained an exemption. Community members uploaded vaccine information including the type of vaccine(s) and dates of vaccinations and boosters as a condition of employment and access to campus. This information was used to determine vaccine status on the date of first positive COVID-19 test. The case investigation interviews also collected data about whether medical care was sought, hospitalizations, and administration of monoclonal antibodies.

### Statistical Analysis

We described continuous variables by using medians and interquartile ranges (IQRs) and categorical variables by using frequencies and percentages. We compared characteristics of survey respondents by using χ^2^ tests for categorical variables and Wilcoxon rank-sum tests for continuous variables. We used logistic regression to determine unadjusted associations between characteristics of survey respondents and long COVID status. We included characteristics that were found to be significantly associated with long COVID status in bivariate analyses in multivariable logistic regression models. All hypothesis tests were 2-sided, and statistical significance was set at an α of 0.05. We performed analyses by using SAS version 9.4 (SAS Institute, Inc., https://www.sas.com).

All university community members provided informed consent to participate in the George Washington University COVID-19 surveillance program. The George Washington University Institutional Review Board concluded that these were non‒research-related activities.

## Results

Overall, the median age of respondents was 23 (IQR 21–32) years, and the median symptom count was 4 (IQR 1–6) (Table 2). More than half of respondents were female (63.4%) and non-Hispanic White (55.7%). Most (73.4%) respondents were students; 26.6% were faculty/staff. The median days from end of isolation to the follow-up survey was 57 (IQR 39–158) days. 

**Table 2:** Characteristics of survey respondents by long COVID status for postacute sequelae of SARS-CoV-2 in university setting, Washington, DC, USA*

Characteristic	Total, n = 1,338	No long COVID, n = 833	Long COVID,† n = 475	p value
Age, y median (IQR)	23 (21–32)	23 (21–33)	23 (21–30)	0.265
Sex, n = 1,327				0.002
F	841 (63.4)	497 (59.1)	344 (40.9)	
M	486 (36.6)	328 (67.5)	158 (31.5)	
Race/ethnicity, n = 1,319				0.089
Non-Hispanic White	734 (55.7)	439 (59.8)	295 (40.2)	
Asian	175 (13.3)	119 (68.0)	56 (32.0)	
Non-Hispanic Black	175 (13.3)	117 (66.9)	58 (33.1)	
Hispanic	117 (8.9)	77 (65.8)	40 (34.2)	
Other	70 (5.3)	37 (52.9)	33 (47.1)	
Multiracial	48 (3.6)	32 (66.7)	16 (33.3)	
Affiliation				0.115
Students	982 (73.4)	599 (61.0)	383 (39.0)	
Faculty/staff	356 (26.6)	234 (65.7)	122 (34.3)	
Underlying conditions‡, n = 1,262				0.003
No	949 (75.2)	613 (64.6)	336 (35.4)	
Yes	313 (24.8)	173 (55.3)	140 (44.7)	
Smoking status, n = 1,259				0.028
Never	1,045 (83.0)	655 (62.7)	390 (37.3)	
Former/current	214 (17.0)	117 (54.7)	97 (45.3)	
Vaccination status at time of positive test result				<0.0001
Fully vaccinated with booster	555 (41.5)	411 (74.1)	144 (25.9)	
Fully vaccinated	400 (29.9)	213 (55.6)	170 (44.4)	
Not fully vaccinated	383 (28.6)	209 (52.3)	191 (47.7)	
Any symptoms at time of positive test result, n = 1,328				<0.0001
No	278 (20.9)	231 (83.1)	47 (16.9)	
Yes	1,050 (79.1)	595 (56.7)	455 (43.3)	
Symptom type at time of positive test result, n = 1,050				
Congestion/cough/sore throat/runny nose	966 (92.0)	549 (56.8)	417 (43.2)	0.713
Headache	538 (51.2)	275 (51.1)	263 (48.9)	0.0002
Fatigue	537 (51.1)	256 (47.7)	281 (52.3)	<0.0001
Chills/measured fever/subjective fever	471 (44.9)	240 (51.0)	231 (49.0)	0.0008
Muscle pain	326 (31.1)	148 (45.4)	178 (54.6)	<0.0001
Chest pain/shortness of breath	189 (18.0)	67 (35.5)	122 (64.5)	<0.0001
Diarrhea/nausea/vomiting	181 (17.2)	78 (43.1)	103 (56.9)	<0.0001
Loss of taste/smell	180 (17.1)	86 (47.8)	94 (52.2)	0.008
Other	59 (5.6)	28 (47.5)	31 (52.5)	0.142
Symptom count, median (IQR)	4 (1–6)	3 (0–5)	5 (3–8)	<0.0001
Sought medical care, n = 1,336				<0.0001
No	1,290 (96.6)	819 (63.5)	471 (36.5)	
Yes	46 (3.4)	12 (26.1)	34 (73.9)	
Received monoclonal antibodies, n = 1,331				0.0012
No	1,258 (94.5)	788 (62.6)	470 (37.4)	
Unknown	48 (3.6)	33 (68.8)	15 (31.2)	
Yes	25 (1.9)	7 (28.0)	18 (72.0)	

*Values are no. (%) except as indicated. IQR, interquartile range. †Long COVID was defined as experiencing >1 of the following symptoms lasting for >28 d after a respondent’s 10-day isolation period ended: difficulty driving, difficulty having conversations, difficulty making decisions, difficulty thinking, fatigue, feeling anxious, feeling depressed or sad, loss of smell, loss of taste, memory loss, muscle pain, muscle weakness, shortness of breath or difficulty breathing, trouble sleeping, worsening of symptoms after physical activity, worsening of symptoms after mental activity, or other symptoms. ‡Includes diabetes, asthma, hypertension, obesity, sickle cell disease, cancer, chronic kidney disease, lung diseases, serious heart conditions, and other conditions.

Most respondents had no underlying conditions (75.2%), never smoked (83.0%), had acute COVID-19 symptoms (79.1%), did not seek medical care at the time of their first positive COVID-19 result (96.6%), and did not receive monoclonal antibody treatment (94.5%) (Table 2). Approximately 41.5% of respondents had received a booster vaccine, 29.9% were fully vaccinated with an initial vaccine series, and 28.6% were not fully vaccinated at the time of their first positive COVID-19 test result. The most common acute symptom was upper respiratory (e.g., congestion, cough, sore throat, runny nose) (92.0%), followed by headache (51.2%), fatigue (51.1%), and chills/fever (44.9%).

Nearly 36% of survey respondents reported experiencing symptoms of long COVID (Table 2). Respondents who had underlying conditions (44.7%; p = 0.003), who were not fully vaccinated (47.7%; p<0.0001), who were female (40.9%; p = 0.002), who were former/current smokers (45.3%; p = 0.028), who experienced acute COVID-19 symptoms (43.3%; p<0.0001), who reported higher symptom counts (mean 5; p<0.0001), who sought medical care (73.9%; p<0.0001), or who received antibody treatment (72.0%; p = 0.0012) were significantly more likely to report symptoms of long COVID. All symptom categories were strongly associated with long COVID status, except for upper respiratory and other symptoms (Table 2; Figure).

**Figure:**
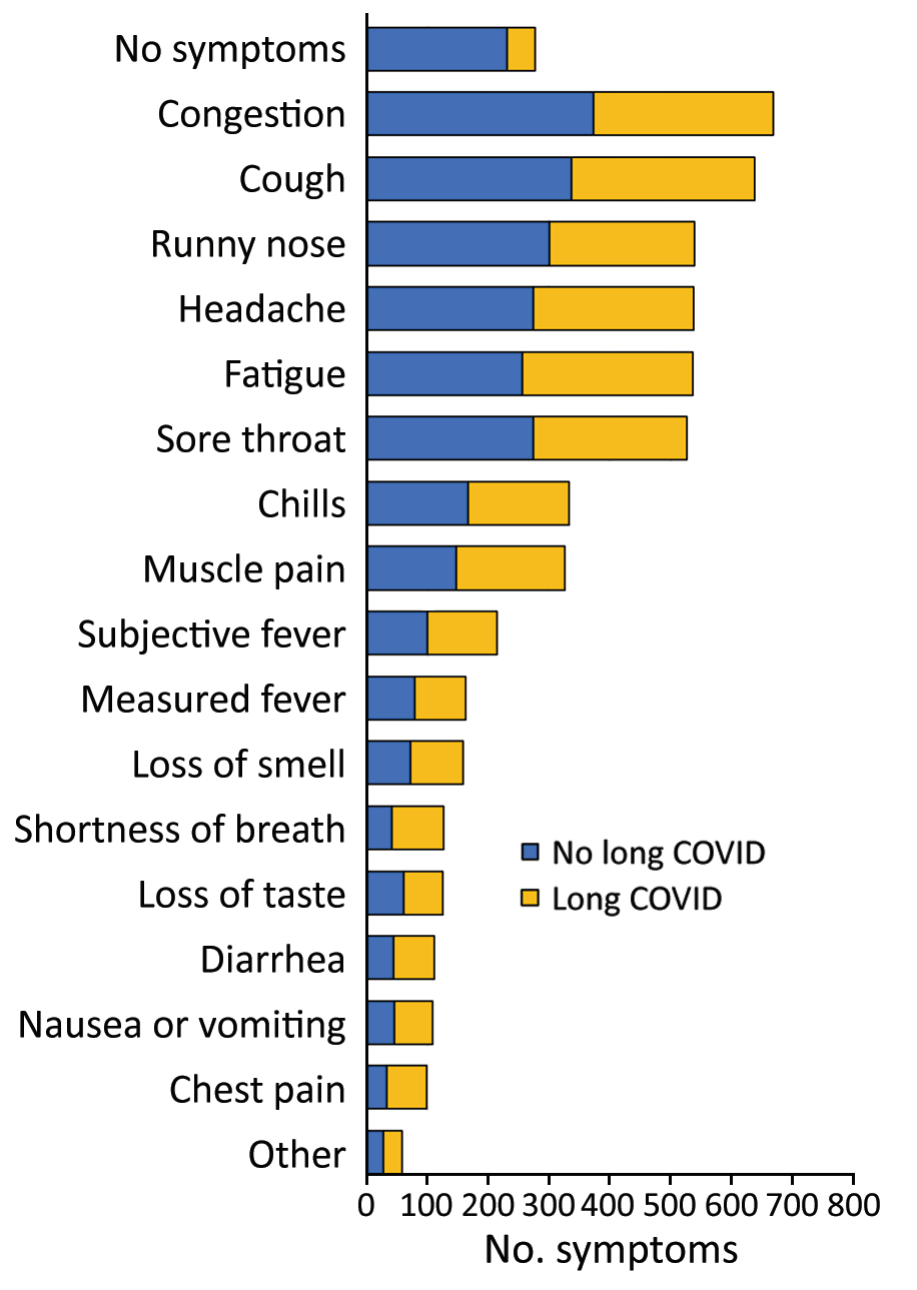
Frequency of reported acute symptoms among survey respondents for postacute sequelae of SARS-CoV-2 in university setting, by long COVID status, Washington, DC, USA (n = 1,338).

Unadjusted associations between characteristics of survey respondents and long COVID status showed that sex, race/ethnicity, underlying conditions, smoking status, vaccination status, any symptoms, symptom type, symptom count, seeking out medical care, and receiving antibody treatment were strongly associated with long COVID (Table 3). Multivariable models adjusting for statistically significant characteristics in the bivariate analyses found several significant associations: smoking history (former/current smokers versus never smokers) (model 1: adjusted odds ratio [aOR] 1.59, 95% CI 1.13–2.25); experiencing any symptoms at the time of positive test (model 1: aOR 1.92, 95% CI 1.01–3.62); experiencing fatigue (model 1: aOR 1.80, 95% CI 1.32–2.47); and experiencing chest pain/shortness of breath (model 1: aOR 2.18, 95% CI 1.48–3.22). Immunization status was significantly associated with long COVID; those fully vaccinated had higher odds of long COVID than those who had also received a booster (model 1: aOR 2.10, 95% CI 1.51–2.90), and those who were not fully vaccinated had higher odds than those fully vaccinated and those given a booster (model 1: aOR 2.71, 95% CI 1.94–3.77). We found similar results after using symptom count in lieu of any symptoms (versus no symptoms) in model 2.

**Table 3:** Association between characteristics of survey respondents and long COVID for postacute sequelae of SARS-CoV-2 in university setting, Washington, DC, USA*

Characteristic	Unadjusted OR (95% CI), n = 1,338	Model 1, aOR† (95% CI), n =1,172	Model 2, aOR† (95% CI), n = 1,175
Age, y	0.99 (0.98–1.00)	‒	‒
Sex, n = 1,327			
F	1.44 (1.14–1.82)	1.22 (0.92–1.62)	1.16 (0.88–1.54)
M	Referent	Referent	Referent
Race/ethnicity, n = 1,319			
Non-Hispanic White	Referent	Referent	Referent
Asian	0.70 (0.49–0.99)	0.97 (0.64–1.46)	1.00 (0.66–1.50)
Non-Hispanic Black	0.74 (0.52–1.04)	0.79 (0.53–1.19)	0.81 (0.54–1.21)
Hispanic	0.77 (0.51–1.16)	0.82 (0.52–1.19)	0.85 (0.53–1.34)
Other	1.33 (0.81–2.17)	1.54 (0.87–2.74)	1.48 (0.84–2.63)
Multiracial	0.74 (0.40–1.38)	0.78 (0.39–1.59)	0.81 (0.40–1.64)
Affiliation			
Students	1.23 (0.95–1.58)	‒	‒
Faculty/staff	Referent	‒	‒
Underlying conditions,‡ n = 1,262			
No	Referent	Referent	Referent
Yes	1.48 (1.14–1.91)	1.23 (0.90–1.66)	1.27 (0.94–1.73)
Smoking status, n = 1,259			
Never	Referent	Referent	Referent
Former/current	1.39 (1.04–1.87)	1.59 (1.13–2.25)	1.60 (1.13–2.25)
Vaccination status at time of positive test result			
Fully vaccinated with booster	Referent	Referent	Referent
Fully vaccinated	2.28 (1.73–3.01)	2.10 (1.51–2.90)	2.19 (1.58–3.03)
Not fully vaccinated	2.61 (1.99–3.43)	2.71 (1.94–3.77)	3.01 (2.16–4.21)
Any symptoms at time of positive test result, n = 1,328			
No	Referent	Referent	‒
Yes	3.76 (2.68–5.26)	1.92 (1.04–3.62)	‒
Symptom type at time of positive test result			
Fatigue	2.83 (2.25–3.56)	1.80 (1.32–2.47)	1.53 (1.05–2.22)
Chest pain/shortness of breath	3.64 (2.64–5.03)	2.18 (1.48–3.22)	1.70 (1.08–2.67)
Congestion/cough/sore throat/runny nose	2.45 (1.87- 3.21)	0.96 (0.58–1.60)	1.07 (0.69–1.65)
Chills/measured fever/subjective fever	2.08 (1.65–2.62)	0.93 (0.69–1.26)	0.73 (0.49–1.08)
Headache	2.21 (1.76–2.77)	1.11 (0.82–1.50)	0.97 (0.69–1.37)
Loss of taste/smell	1.99 (1.45–2.73)	1.04 (0.72–1.49)	0.83 (0.53–1.29)
Muscle pain	2.52 (1.95–3.25)	1.25 (0.91–1.73)	1.10 (0.78–1.57)
Diarrhea/nausea/vomiting	2.48 (1.80–3.41)	1.24 (0.85–1.81)	1.02 (0.66–1.56)
Other	1.88 (1.11–3.17)	0.89 (0.48–1.64)	0.83 (0.44–1.55)
Symptom count	1.22 (1.18–1.27)	‒	1.16 (1.00–1.33)
Sought out medical care, n = 1,336			
No	Referent	Referent	Referent
Yes	4.93 (2.53–9.61)	2.17 (0.98–4.77)	2.07 (0.94–4.55)
Received monoclonal antibodies, n = 1,331			
No	Referent	Referent	Referent
Unknown	0.76 (0.41–1.42)	0.74 (0.34–1.64)	0.72 (0.33–1.59)
Yes	4.31 (1.79–10.40)	1.93 (0.63–5.96)	2.06 (0.68–6.23)

*Long COVID was defined as experiencing >1 of the following symptoms lasting for >28 d after a respondent’s 10-day isolation period ended: difficulty driving, difficulty having conversations, difficulty making decisions, difficulty thinking, fatigue, feeling anxious, feeling depressed or sad, loss of smell, loss of taste, memory loss, muscle pain, muscle weakness, shortness of breath or difficulty breathing, trouble sleeping, worsening of symptoms after physical activity, worsening of symptoms after mental activity, or other symptoms. aOR, adjusted odds ratio; IQR, interquartile range; OR, odds ratio; ‒, variable omitted for that model. †Adjusted for all other variables in the column. ‡Includes diabetes, asthma, hypertension, obesity, sickle cell disease, cancer, chronic kidney disease, lung diseases, serious heart conditions, and other conditions.

## Discussion

This study aimed to examine the prevalence and predictors of long COVID in a university community. This sample was unique in that it consisted of primarily young adults who had few underlying health conditions and otherwise were considered healthy. Regardless of initial symptoms, nearly 36% of COVID-19 survivors in this study reported experiencing symptoms consistent with long COVID. That result is within ranges found in other studies reporting a prevalence of long COVID of anywhere from 10% to 80% among COVID-19 survivors (*3**–**5*,*7*,*21*,*29**–**31*). Our study also found an increased odds of reporting symptoms consistent with long COVID for each additional symptom reported during the initial infection. This finding is consistent with recent studies conducted with a high proportion of young adults that also found a higher number of acute symptoms during a COVID-19 infection predicted >1 long COVID symptom (*32*). Monitoring symptoms of initial cases could help identify persons at risk for long COVID.

Our study also found that persons who had the fewest previous COVID-19 vaccines and boosters were at higher risk for development of symptoms consistent with long COVID, supporting other investigations suggesting that vaccination is associated with reduced risk for long COVID (*33**–**36*). Many colleges and universities required the COVID-19 vaccine before the fall 2021 semester but offered reasonable medical/religious exemptions. Our results further highlight the need for routine short- and long-term follow-up for persons who test positive for COVID-19 while continuing to advocate and monitor for vaccine and booster adherence to published recommendations.

Although prevention efforts are needed for long COVID, the findings from this study support the need to ameliorate consequences of long COVID. Based on symptomatology, recovery strategies for long COVID include physical rehabilitation, management of preexisting conditions, mental health support, social services support, and exercise programs scaled to the ability of the patient (*11*,*37*). Because long COVID can greatly interfere with the ability to learn or work, classroom or job accommodations, such as modifying academic and workplace policies, flexible scheduling, changing workplace environment, enabling remote or alternative learning, and modifying job responsibilities, are recommended for those having long COVID.

Limitations in conducting this study included the possibility of recall bias, loss to follow-up, and digital literacy challenges, as well as acknowledgment that the results are only for persons who tested positive for SARS-CoV-2. Persons were asked to recall information about their illness after >28 days had passed. Considering brain fog is a symptom consistent with long COVID and the length of time between isolation and follow-up, some persons who had long COVID might have forgotten details of their health status during a tumultuous time in their life. Although inevitable, this situation was mitigated by providing the person with dates of their illness when asking them to think back to that time.

Loss to follow-up was also a limitation; some persons never completed a case investigation, which made it more likely for them to forgo a follow-up months later. CCST made >3 attempts at different time points throughout the day to reach as many persons as possible. Those strategies, and our achieved response rate, are consistent with other COVID-19 studies conducted during the pandemic (*38*). Nonetheless, we acknowledge that results could be inflated because persons experiencing symptoms consistent with long COVID might be more likely to respond. Thus, results should be interpreted with caution.

In addition, surveys were conducted by electronic survey and telephone. Although there were no major differences in demographics between telephone and electronic survey completion, some of our participants did not have smartphones, only had landlines, or could not be reached by email, which contributed to loss to follow-up.

Finally, our sample was only of persons who had COVID-19 within our campus community and not of the entire campus population. Thus, it is not possible to know whether symptoms reported in our survey were also increased in the campus population as a whole during this time. Many of the symptoms in our survey are common and might or might not be directly related to SARS-CoV-2 infection or long COVID.

Public health experts and healthcare providers have been gathering data about COVID-19 while simultaneously trying to understand the long-term consequences of SARS-CoV-2 infection. Although preliminary findings of long COVID were anecdotal, researchers continue to gain a clearer picture on who it affects and how it affects certain populations. From a university standpoint, this analysis is key to understanding how administration can fill the needs of the campus population that has long-term complications caused by COVID-19. Paired with the recommendations presented in this article, universities can strengthen existing services or make referrals to prevention and rehabilitation services (i.e., mental health, exercise programs, long-term health studies) for those who have long COVID that affects their ability to engage in university activities such as classes and work. In addition, universities might benefit from adopting preventive resources for their populations, as well as extended pandemic leave, given the considerable long-lasting effects of long COVID.

Future research avenues should consider following up with long COVID survivors/patients to assess long-term or long-lasting symptoms. Such analysis could explore the consequences of long COVID for 5‒10 years after the initial infection, especially to gain a better understanding of its effect on young, healthy populations. Follow-up could also occur with older populations to assess whether symptoms progress into retirement age and to determine the cost of long-term care resulting from long COVID. Furthermore, research should continue to examine the effect vaccine booster doses have on long COVID symptoms. Such research is vital to clarifying long-term effects of long COVID and how universities can support those dealing with long COVID to promote health and wellness across campus communities.

Appendix 1 Additional information on postacute sequelae of SARS-CoV-2 in university setting.

Appendix 2 COVID-19 follow-up survey for postacute sequelae of SARS-CoV-2 in university setting.
