# Supplementary material for: Postacute Sequelae of SARS-CoV-2 in University Setting
Source: Emerg Infect Dis. 2023 Mar;29(3):519–27. doi: 10.3201/eid2903.221522 (PMC9973677; doi:10.3201/eid2903.221522)
Supplement: Appendix 1 — Additional information on postacute sequelae of SARS-CoV-2 in university setting. [file 22-1522-Techapp-s1.pdf]

Article DOI: https://doi.org/10.3201/eid2903.221522

Postacute Sequelae of SARS-CoV-2 in University Setting

Appendix 1.

Appendix 1 Table. Characteristics of follow-up survey respondents versus nonrespondents with a completed case interview*

Characteristic	Total, no. (%), (n = 3,595)	Nonrespondents, no. (%), (n = 2,254)	Respondents, no. (%), (n = 1,341)	p value
Age, y, median (IQR)	22 (20–27)	22 (20–26)	23 (21–32)	<0.0001
Sex (n = 3,546)				0.0464
Female	2,173 (61.3)	1,330 (60.0)	834 (63.4)	
Male	1,373 (38.7)	886 (39.9)	487 (36.6)	
Race/Ethnicity (n = 3,538)				0.9185
Non-Hispanic White	1,986 (56.1)	1,251 (56.5)	735 (55.6)	
Asian	464 (13.1)	289 (13.9)	175 (13.2)	
Non-Hispanic Black	443 (12.5)	268 (12.1)	175 (13.2)	
Hispanic	314 (8.9)	195 (8.8)	119 (9.0)	
Other	196 (5.4)	126 (5.7)	70 (5.3)	
Multiracial	135 (3.8)	87 (3.9)	48 (3.6)	
Affiliation				<0.0001
Student	2,930 (81.5)	1,946 (86.3)	984 (73.4)	
Faculty/staff	665 (18.5)	308 (13.7)	357 (26.6)	
Any underlying condition† (n = 3,383)				<0.0001
No	2,668 (78.9)	1,717 (81.1)	951 (75.2)	
Yes	715 (21.1)	401 (18.9)	314 (24.8)	
Smoking status				0.7135
Never	2,876 (83.1)	1,794 (82.9)	1,082 (83.4)	
Current/former	584 (16.9)	369 (17.1)	215 (16.6)	
Vaccination status at time of test (n = 3,592)				<0.0001
Fully vaccinated with booster	1,404 (39.1)	849 (37.7)	555 (41.4)	
Fully vaccinated	1,319 (36.7)	935 (41.5)	384 (28.6)	
Not fully vaccinated	869 (24.2)	467 (20.8)	402 (29.9)	
Any symptoms at time of test‡ (n = 3,574)				0.8788
No	754 (21.1)	475 (21.2)	279 (20.9)	
Yes	2,820 (78.9)	1,768 (78.8)	1,052 (79.0)	
Symptom count, mean (IQR)	4 (1–6)	4 (1–6)	4 (1–6)	0.9871

*IQR, interquartile range.
†Includes diabetes, asthma, hypertension, obesity, sickle cell disease, cancer, chronic kidney disease, lung diseases, serious heart conditions, and other conditions.
‡Includes chest pain, chills, congestion, cough, diarrhea, fatigue, fever, headache, loss of smell, loss of taste, muscle pain, nausea or vomiting, runny nose, shortness of breath, sore throat, and other symptoms.
