# Supplementary material for: Postacute Sequelae of SARS-CoV-2 in University Setting
Source: Emerg Infect Dis. 2023 Mar;29(3):519–27. doi: 10.3201/eid2903.221522 (PMC9973677; doi:10.3201/eid2903.221522)
Supplement: Appendix 2 — COVID-19 follow-up survey for postacute sequelae of SARS-CoV-2 in university setting. [file 22-1522-Techapp-s2.pdf]

Article DOI: https://doi.org/10.3201/eid2903.221522

EID cannot ensure accessibility for supplementary materials supplied by authors. Readers who have difficulty accessing supplementary content should contact the authors for assistance.

Postacute Sequelae of SARS-CoV-2 in University Setting

Appendix 2

The following pages show the script for the long COVID survey, a follow-up telephone interview given to 4,800 persons with COVID-19 cases identified by The George Washington University COVID-19 surveillance and testing program during August 2020–February 2022. The surveys were administered by The George Washington University Campus COVID-19 Support Team during July 2021–March 2022.

COVID-19 Follow-up Survey

Dear [first_name],

Thank you for participating in this confidential survey. We are aiming to better understand the potential long-term effects and health behaviors surrounding COVID-19 in the GWU community. All of your responses will be grouped together for any reporting purposes and your identity will remain confidential. This survey should take 5-10 minutes to complete.

Thank you for your time!

This set of questions asks about your experience during the two weeks after you tested positive for COVID-19.

SCRIPT:

This first set of questions asks about symptoms you may have experienced DURING the two weeks after you tested positive.

I see that you tested positive for COVID-19 on [lab_pos_date]. I am going to run through a list of symptoms quickly. Please say "yes" for any you experienced during the two weeks after you tested positive.

You tested positive for COVID-19 on [lab_pos_date] (m/d/y). Thinking back to that time, did you experience any of the following symptoms during the two weeks after you tested positive?
(Select all that apply)

- ☐ Difficulty driving
- ☐ Difficulty having conversations
- ☐ Difficulty following instructions
- ☐ Difficulty making decisions
- ☐ Difficulty thinking (known as "brain fog")
- ☐ Fatigue
- ☐ Feeling anxious
- ☐ Feeling depressed or sad
- ☐ Loss of smell
- ☐ Loss of taste
- ☐ Memory loss
- ☐ Muscle pain
- ☐ Muscle weakness
- ☐ Shortness of breath or difficulty breathing
- ☐ Trouble sleeping
- ☐ Worsening of symptoms after physical activity (e.g., walking, swimming, running, etc.)
- ☐ Worsening of symptoms after mental activity (e.g., work, attending school)
- ☐ Other, please specify
- ☐ No symptoms

Other symptoms: ______________________

Of the symptoms selected, which symptom was the most bothersome to you ?<br>(Choose only one symptom)	◯ Difficulty driving<br>◯ Difficulty having conversations<br>◯ Difficulty following instructions<br>◯ Difficulty making decisions<br>◯ Difficulty thinking (known as "brain fog")<br>◯ Fatigue<br>◯ Feeling anxious<br>◯ Feeling depressed or sad<br>◯ Loss of smell<br>◯ Loss of taste<br>◯ Memory loss<br>◯ Muscle pain<br>◯ Muscle weakness<br>◯ Shortness of breath or difficulty breathing<br>◯ Trouble sleeping<br>◯ Worsening of symptoms after physical activity (e.g., walking, swimming, running, etc.)<br>◯ Worsening of symptoms after mental activity (e.g., work, attending school)<br>◯ Other, please specify
Other symptoms:	__________________________________
After you tested positive for COVID-19, did you ever have to seek medical care in the emergency room or urgent care?	◯ Yes<br>◯ No
After you tested positive for COVID-19, were you ever hospitalized (i.e., had to stay overnight in the hospital)?	◯ Yes<br>◯ No
Did you receive any monoclonal antibodies to treat your COVID-19? (Antibodies assist the immune system to respond more effectively to the virus.)	◯ Yes<br>◯ No<br>◯ Unknown
Do you have any of the following conditions?<br>(Select all that apply)	☐ Alcohol or substance use disorder<br>☐ Asthma<br>☐ Cancer<br>☐ Cardiovascular diseases (heart attack/failure, stroke, etc.)<br>☐ Chronic kidney diseases<br>☐ Chronic lung diseases (COPD)<br>☐ Depression<br>☐ Diabetes<br>☐ Hepatitis B virus (HBV)<br>☐ Hepatitis C virus (HCV)<br>☐ HIV<br>☐ Hypertension (high blood pressure)<br>☐ Tuberculosis (TB)<br>☐ Other mental health condition<br>☐ Other chronic condition (specify)<br>☐ No underlying conditions
Other conditions:	__________________________________
Are you currently pregnant?	◯ Yes<br>◯ No

SCRIPT:

Thank you for that information.

The next set of questions are about behaviors before you tested positive and during your 10-day isolation period. The answers to these questions are on a scale from...
(READ SLOWLY)

- Rarely or none of the time
- Some or little of the time
- Occasionally or a moderate amount of time
- Most or all of the time

The next set of questions asks about behaviors before you tested positive and during your 10-day isolation period.

	Rarely or none of the time	Some or little of the time	Occasionally or a moderate amount of time	Most or all of the time
BEFORE becoming infected, how often did you practice social distancing? (social distancing = staying 3-6 feet away from other people when out in public or in social settings such as work or school)	○	○	○	○
BEFORE becoming infected, how often did you consistently wear any PPE (masks, N95, face shields, etc.) when around others outside of your immediate household members?	○	○	○	○
BEFORE testing positive, how often did you stay at home as much as possible?	○	○	○	○
AFTER you tested positive, how often did you self-isolate or quarantine for the recommended/required number of days? (i.e., you separated yourself from other people, even those in your own household, to prevent others from getting sick)	○	○	○	○

The next set of questions asks about symptoms you may have experienced after your 10-day isolation period ended.

SCRIPT:

The next set of questions are about symptoms AFTER your 10-day isolation period ENDED.

Please think back to the time AFTER your isolation ENDED. Have you had any symptoms that lasted more than 28 days? I am going to run through a list of symptoms quickly again. Please say "yes" for any you experienced during the two weeks after you tested positive.

AFTER your 10-day isolation period ENDED, have you had any of the following symptoms [lasting for more than 28 days after your 10-day isolation period ended]?
(Select all that apply)

- ☐ Difficulty driving
- ☐ Difficulty having conversations
- ☐ Difficulty following instructions
- ☐ Difficulty making decisions
- ☐ Difficulty thinking (known as "brain fog")
- ☐ Fatigue
- ☐ Feeling anxious
- ☐ Feeling depressed or sad
- ☐ Loss of smell
- ☐ Loss of taste
- ☐ Memory loss
- ☐ Muscle pain
- ☐ Muscle weakness
- ☐ Shortness of breath or difficulty breathing
- ☐ Trouble sleeping
- ☐ Worsening of symptoms after physical activity (e.g., walking, swimming, running, etc.)
- ☐ Worsening of symptoms after mental activity (e.g., work, attending school)
- ☐ Other, please specify
- ☐ No symptoms

Other symptoms: ______________________________

Have you had to reduce your hours at work or at school due to persistent COVID-19 symptoms AFTER your 10-day isolation period?

- ◯ Yes
- ◯ No

Are you still having any COVID-19 related symptoms?

- ◯ I have recovered and am symptom free
- ◯ I am feeling better but not completely recovered (i.e., have COVID-19 related symptoms that remain)
- ◯ I am not feeling better

SCRIPT:

The answers to the next question are on a scale from...

- Not at all impacted
- A little impacted
- Moderately impacted
- Extremely impacted

Overall, considering all the possible ways your life may have been affected because of persistent COVID-19 symptoms, how much has this impacted your day-to-day life?

- ◯ Not at all impacted
- ◯ A little impacted
- ◯ Moderately impacted
- ◯ Extremely impacted

Question	Response
Have you received the COVID-19 vaccine?	◯ Yes<br>◯ No
Are you fully vaccinated? (e.g., 1 dose of J&J or 2 doses of AstraZeneca/Moderna/Pfizer/etc.)	◯ Yes<br>◯ No
Did your symptoms improve at all after receiving the vaccine?	◯ Yes<br>◯ No

The final set of questions asks about the impact COVID-19 has had on you, family, and friends.

SCRIPT:

The answers to the next question are on a scale from...

- Not at all concerned
- A little concerned
- Moderately concerned
- Extremely concerned

Question	Response
How concerned are you about becoming re-infected with COVID-19?	◯ Not at all concerned<br>◯ A little concerned<br>◯ Moderately concerned<br>◯ Extremely concerned
How many people do you know who have been diagnosed with COVID-19 ? ______ people	______________________________
We are interested in learning more about the lasting effects of COVID-19 among the GWU population. May we contact you for additional information about your experience?	◯ Yes<br>◯ No

SCRIPT:

Thank you for participating in this confidential survey. As a reminder...

- Everything that we talk about will be kept confidential.
- Your responses will be grouped with others to hide your identity.
- This information will not impact your GW status.
- The information you've shared will be very helpful in understanding the long term effects of COVID-19.
- You will receive an email with a list of resources if you or someone else needs them.

Do you have any final questions?

Thank you for your time!

*****IMPORTANT*****

Select: SAVE & MARK SURVEY AS COMPLETE (Do not press SAVE & EXIT FORM)
